# Sensorineural hearing loss in anti-interleukin-1 treated CAPS patients: risk factors and real-life barriers—an observational study

**DOI:** 10.1093/rheumatology/keaf355

**Published:** 2025-08-01

**Authors:** Özlem Satirer, Anke Tropitzsch, Assen Koitschev, Toni Hospach, Susanne M Benseler, Jasmin B Kuemmerle-Deschner

**Affiliations:** Paediatric Rheumatology, Department of Paediatrics and autoinflammation reference center Tuebingen (arcT), University Hospital Tuebingen, Tuebingen, Germany; Department of Otorhinolaryngology, Head and Neck Surgery, University Hospital Tuebingen, Tuebingen, Germany; Department of Otorhinolaryngology, Head and Neck Surgery, Klinikum Stuttgart, Stuttgart, Germany; Department of Paediatrics, Paediatric Rheumatology, Klinikum Stuttgart, Stuttgart, Germany; Rheumatology, Department of Paediatrics, Alberta Children's Hospital (ACH), ACH Research Institute, Cumming School of Medicine, University of Calgary, Calgary, AB, Canada; Children’s Health Ireland, University College Dublin & Trinity College, Dublin, Ireland; Paediatric Rheumatology, Department of Paediatrics and autoinflammation reference center Tuebingen (arcT), University Hospital Tuebingen, Tuebingen, Germany

**Keywords:** NLRP3 mutation, cryopyrin-associated periodic syndrome, hearing loss, inner ear, pure tone average, high frequency

## Abstract

**Objective:**

To identify modifiable risk factors associated with progression of hearing impairment from a longitudinal cohort of anti-IL-1-treated children and adults with Cryopyrin-Associated Periodic Syndromes (CAPS) and explore real-life barriers to optimal long-term management.

**Methods:**

A single-centre, longitudinal study included consecutive paediatric and adult anti-IL-1-treated CAPS patients with sensorineural hearing loss between 2006 and 2024. Data collected encompassed demographics, disease characteristics, genotype, treatment regimens and hearing assessments using 4PTA, HF-PTA. Primary outcome was WHO grade of hearing impairment at last follow-up. Factors associated with hearing impairment and real-life barriers mandating therapy escalation were identified.

**Results:**

The study included 36 patients; 20 males, 16 females, median age at CAPS disease onset and hearing loss diagnosis was 11.8 and 40 years, respectively. Most patients (83%) exhibited moderate CAPS phenotype, carrying pathogenic or likely pathogenic NLRP3 variants (78%). Hearing loss was present in 83% at baseline and 88% at last follow-up with HF-PTA-sensitivity of 100%. Patients diagnosed in adulthood, those with a late treatment start, and/or with pathogenic or likely pathogenic variants demonstrated higher WHO grades of hearing impairment. Ten patients required therapy escalation due to progressive hearing loss, eight of whom carried pathogenic mutations. Early progression was primarily driven by disease activity, while late progression was predominantly influenced by non-compliance. Over time, 86% maintained stable hearing, 8% showed improvement and 6% experienced worsening.

**Conclusion:**

Early diagnosis, timely intervention and a refined Treat-to-Target approach are vital for hearing in lifelong CAPS management. Precision care and continuous monitoring are key to improving long-term outcomes.

Rheumatology key messages

- Early diagnosis, including HF-PTA, and start of anti-IL-1 therapies are essential for optimal CAPS outcomes.
- Severe hearing impairment is linked with adult-onset, delayed treatment (>10 years), pathogenic variants and female sex.
- Late hearing loss progression highlights the need for a refined long-term treat-to-target approach in CAPS.

## Introduction

Cryopyrin-Associated Periodic Syndromes (CAPS) are rare autoinflammatory diseases characterized by dysregulated activation of NLRP3 inflammasome and increased production of interleukin-1 (IL-1) beta, the main mediator of innate inflammation [1, 2]. Continuous inflammation can result in organ system dysfunction, leading to significant morbidity and long-term complications such as amyloidosis, and sensorineural hearing loss [3] the latter having a profound impact on health related quality of life as captured in the World Health Organization (WHO) classification of hearing impairment [4]. Anti-IL-1 therapy in CAPS was shown to effectively control systemic inflammation and prevent organ dysfunction [5]. Treat-to-Target (T2T) approaches aim to maximize the effectiveness of anti-IL-1 therapy for individual patients.

In CAPS, sensorineural hearing loss typically begins in childhood or early adulthood and often progresses insidiously, initially manifesting as high-frequency hearing loss. [6]. This early stage is often missed due to the lack of inclusion of high frequencies in standard audiological evaluations; a designated testing protocol including these frequencies was proposed [6, 7]. Emerging evidence suggests that early intervention with IL-1 blockers in CAPS patients may partially reverse inner ear inflammation and improve hearing outcomes [5, 8].

However, evidence of long-term outcomes of targeted therapies, particularly those aimed at preserving hearing, in the lifelong illness CAPS remain limited. Identifying factors associated with an increased risk of hearing loss progression may serve as guidance for long-term monitoring protocols. In addition, real-life barriers towards optimal, personalized treatment strategies for children and adults living with CAPS-related hearing loss need to be detected.

Therefore, the aims of study were: (1) to describe a longitudinal cohort of consecutive anti-IL1-treated paediatric and adult CAPS patients with sensorineural hearing loss, (2) to examine risk factors associated with a high WHO grade of hearing impairment and (3) to identify real-life barriers to optimal long-term management of hearing loss in CAPS.

## Materials and methods

A single-centre study of consecutive paediatric and adult CAPS patients with sensorineural hearing loss diagnosed between January 2006 and June 2024 was performed. Inclusion criteria were: (1) meeting CAPS classification/diagnostic criteria [8–11], (2) having a formal diagnosis of sensorineural hearing loss at diagnosis and/or at any point during the observation period and (3) having complete clinical and audiological data available for analysis. Patients were excluded if (1) they had an unrelated medical condition resulting in hearing impairment and/or (2) were seen only for a second opinion visit at the centre only. The study was approved by the Clinical Ethics Committee at the University Hospital Tuebingen (REB No: 050/2021BO2) and informed consent was provided for the publication of this article.

### Data

Patient- and disease-related information included gender, age at disease onset, age at diagnosis, age at hearing loss diagnosis, age at the start of treatment, time to and type of treatment escalation and time from symptom onset to treatment initiation. The latter was dichotomized into early (<10 years) and late treatment start (≥10 years) as published previously [12]. CAPS phenotype (FCAS, MWS, CINCA/NOMID) and NLRP3 variants including classification according to the American College of Medical Genetics and Genomics were recorded [13]. Disease activity was captured using the previously reported Physician Global assessment (PGA) and Patient/Parent Global Assessment (PPGA) on a 10 cm Visual Analogue Scale categorized as mild (<2), moderate (2–4) and high (>4) [14]. Disease activity was defined as completely or partially controlled according to established criteria from prior studies [14]. Factors contributing to increased disease activity, including therapy non-compliance, puberty and treatment interruptions due to pregnancy, infections or limited access, were documented.

### Hearing assessment tools

#### Pure Tone Average—4PTA

At each study visit, Pure Tone Averages (PTAs) were determined including 0.5, 1, 2 and 4 kHz for each ear, and compared with normative data. Thresholds ≥10 dB above normative levels were considered abnormal. Normative values were based on age- and frequency-matched groups as previously reported [6].

#### High frequency pure tone average—HF-PTA

HF-PTA was calculated by averaging thresholds at 6 and 8 kHz. Correspondingly, abnormal values were defined as ≥10 dB above normative levels [6].

#### WHO grades of hearing impairment

Hearing loss-related functional disability was classified by WHO criteria based on the 4PTA of the better-hearing ear: Grade 0 (normal, <20 dB), Grade 1 (mild, 20 to <35 dB), Grade 2 (moderate, 35 to <50 dB), Grade 3 (moderately severe, 50 to <65 dB), Grade 4 (severe, 65 to <80 dB), Grade 5 (profound, 80–95 dB) and Grade 6 (total deafness, ≥95 dB) [6, 15]. WHO grade was assessed at baseline, at the therapy escalation visit and at last follow-up.

#### Hearing loss progression timing

Hearing loss progression was dichotomized based on the time to worsening; *early progression* defined as progression within 3 years or less receiving anti-IL1 therapy treatment and *late progression* as progression after more than 3 years on treatment.

#### Treatments

CAPS anti-IL1 therapy: adults received canakinumab 150 mg, and children <40 kg received 2 mg/kg, both subcutaneously every 8 weeks. Anakinra was given at 100 mg/day for patients ≥40 kg, or 1–2 mg/kg/day for those <40 kg, daily. Treatment escalation to control disease activity and/or prevent the progression of hearing loss involved increasing the dose or shortening the interval of canakinumab, increasing the dose of anakinra or using an anakinra–canakinumab combination [11]. Paediatric weight-based escalations were not considered as treatment escalations.

#### Outcomes

The primary outcome was the WHO grade of hearing impairment at the last follow-up visit, which refers to the final audiological evaluation conducted during the study period. Secondary outcomes included: (1) WHO grades of hearing impairment at baseline, defined as the first audiological evaluation upon entering care at the centre, and therapy escalation, (2) disease activity assessed at each study visit and (3) barriers to optimal long-term management of hearing loss.

### Analysis

All statistical analyses were performed using SPSS version 28.0.1.1 (IBM Corp., Armonk, NY, USA) and Stata version 17.0 (StataCorp LLC, College Station, TX, USA). Descriptive statistics summarized demographic and clinical characteristics. Categorical variables were reported as frequencies and percentages; continuous variables as means with standard deviations (S.D.) or medians with ranges, depending on data distribution. Normality was assessed using the Shapiro–Wilk and Kolmogorov–Smirnov tests. For group comparisons, appropriate statistical tests were selected based on data type and distribution: paired *t*-tests or Wilcoxon signed-rank tests for paired data, independent *t*-tests or Mann–Whitney *U* tests for unpaired data, and *χ*^2^ or Fisher’s exact tests for categorical variables. Missing data were handled using complete case analysis; no imputation was performed. All tests were two-sided, with *P* < 0.05 considered statistically significant. Due to the exploratory design and small sample size typical for rare diseases, no correction for multiple testing was applied. Results were interpreted cautiously and in line with clinical relevance. No *a priori* power calculation was conducted, as the study included all available patients meeting predefined eligibility criteria (January 2006 to June 2024), representing a real-life cohort of this rare disease.

## Results

The study included 36 anti-IL1-treated paediatric and adult CAPS patients with sensorineural hearing loss, including 20 males and 16 females. The median age at disease onset was 11.8 years (range: 6 months–50 years) and at CAPS diagnosis 35 years (range: 6 months–75 years). The median age at formal diagnosis of hearing loss was 40 years (range: 6–75 years). The median time from CAPS symptom onset to treatment was 24.8 years (range: 6 months–71 years). The median follow-up duration was 8.2 years (range: 1–18 years).

Thirty patients (83%) presented with the moderate CAPS phenotype, while six patients (17%) exhibited the severe phenotype. Genetic analysis revealed pathogenic NLRP3 variants in 14 patients (39%), including T348M (16%), A439V (14%), D303N (3%), M664T (3%) and R262W (3%). Likely pathogenic variants were detected in 14 patients (39%), with E311K (30%), Y859S (6%) and H358R (3%) while variants of uncertain significance (VUS) were found in five patients (14%), including V198M (8%), Q703K (3%) and N654S (3%). Three patients (8%) had no detectable NLRP3 variants. Childhood-onset disease was observed in 60% (A439V), 64% (E311K) and 83% (T348M), while all patients without a detectable mutation (*n* = 3) had adult-onset disease. Moderate to profound hearing loss was present in 40% (A439V), 91% (E311K) and 83% (T348M) both at baseline and last visit.

At disease onset, 12 (34%) showed severe disease activity, 24 (66%) exhibited moderate disease activity and no patient had mild disease activity as per PGA/VAS definition. Initial treatment consisted of anakinra in 16 patients (44%) and canakinumab in 20 patients (56%). At last follow-up, eight patients (21%) were on anakinra, 27 (71%) on canakinumab and one (3%) on combination therapy with canakinumab and anakinra.

At baseline and serial follow-ups, sensorineural hearing loss was formally evaluated in all patients. Sensorineural hearing loss was present in 30 patients (83%) at baseline and 32 (88%) at last follow-up. Abnormal HF-PTA findings were noted in 59 ears (82%) of the 30 patients at baseline. At last follow-up, 61 ears (85%) of 32 patients were abnormal. Among 36 patients with a total of 72 ears, abnormal 4PTA findings were observed at baseline in 22 patients (61%) affecting 42 ears (58%). At last follow-up, these findings were present in 22 patients (61%) affecting 42 ears (58%). The sensitivity of 4PTA for detecting hearing loss was 71% at baseline and 68% at follow-up, whereas HF-PTA demonstrated 100% sensitivity at both time points (Table 1). Between baseline and last follow-up, 86% of patients had stable WHO grades, 8% improved and 6% worsened. Changes were confirmed by PTA and HF-PTA. SAA levels were not significantly different between hearing outcome groups (*P* = 0.372), and no relevant diagnosis of amyloidosis was observed.

**Table 1.** Baseline characteristics and audiological findings in CAPS patients with sensorineural hearing loss

General characteristics	*N* = 36	
Gender (male:female)	20:16	
Age at disease onset in years (median, range)	11.8 (0–50)	
Age at diagnosis in years (median, range)	35 (0–75)	
Age at hearing loss diagnosis in years (median, range)	40 (6–75)	
Time from symptom onset to treatment in years (median, range)	24.8 (0–71)	
Follow-up in years (median, range)	8.2 (1–18)	
CAPS phenotypes		
Mild phenotype (FCAS), *n* (%)	0 (%)	
Moderate phenotype (MWS), *n* (%)	30 (83%)	
Severe phenotype (CINCA/NOMID), *n* (%)	6 (17%)	
Genetic findings (*NLRP3* variants)		
**Pathogenic, *n* (%)**	**14 (39%)**	
T348M	6 (16%)	
A439V	5 (14%)	
D303N	1 (3%)	
M664T	1 (3%)	
R262W	1 (3%)	
**Likely pathogenic, *n* (%)**	**14 (39%)**	
E311K	11(30%)	
Y859S	2 (6%)	
H358R	1 (3%)	
**Variant of uncertain significance, *n* (%)**	**5 (14%)**	
V198M	3 (8%)	
Q703K	1 (3%)	
N654S	1 (3%)	
No variant detected, *n* (%)	3 (8%)	
Disease activity at diagnosis		
Mild, *n* (%)	0 (0%)	
Moderate, *n* (%)	24 (66%)	
Severe, *n* (%)	12 (34%)	
Initial treatment type		
Anti-IL1 therapy	36 (100%)	
Anakinra, *n* (%)	16 (44%)	
Canakinumab, *n* (%)	20 (56%)	
Audiological findings (sensorineural hearing loss)	Baseline	Last follow-up
Patients with hearing loss, *n* (%)	30 (83%)	32 (88%)
Number of ears evaluated	72	72
Ears with abnormal 4PTA, *n* (%)	42 (58%)	42 (58%)
Median 4PTA (range)	34.5 (0–64.2)	38.5 (0–65)
Ears with abnormal HF-PTA, *n* (%)	59 (82%)	61 (85%)
Median HF-PTA (range)	54.5 (0–120)	55 (0–120)
Sensitivity of 4PTA for detection of hearing loss	71%	68%
Sensitivity of HF-PTA detection of hearing loss	100%	100%

Sensitivity for each method was calculated as follows: 4PTA sensitivity = (number of ears with abnormal 4PTA in patients with hearing loss)/(total number of ears from patients with hearing loss). HF-PTA sensitivity = (number of ears with abnormal HF-PTA in patients with hearing loss)/(total number of ears from patients with hearing loss).

Bold text is used to emphasize findings considered particularly relevant or important for clinical interpretation. These highlighted results are intended to draw attention to the most meaningful aspects of the data.

### Association of patient- and treatment-related factors with hearing outcomes

#### Age at diagnosis

At baseline, hearing impairment was significantly more frequent and severe in patients diagnosed in adulthood (*χ*^2^, *P* = 0.006). All with normal hearing (<20 dB) were diagnosed in childhood, while 78% with severe and 100% with profound loss were diagnosed in adulthood. A significant negative correlation supported this (*r* = −0.46, 95% CI −0.68 to −0.19, *P* = 0.005).

**Figure 1.**
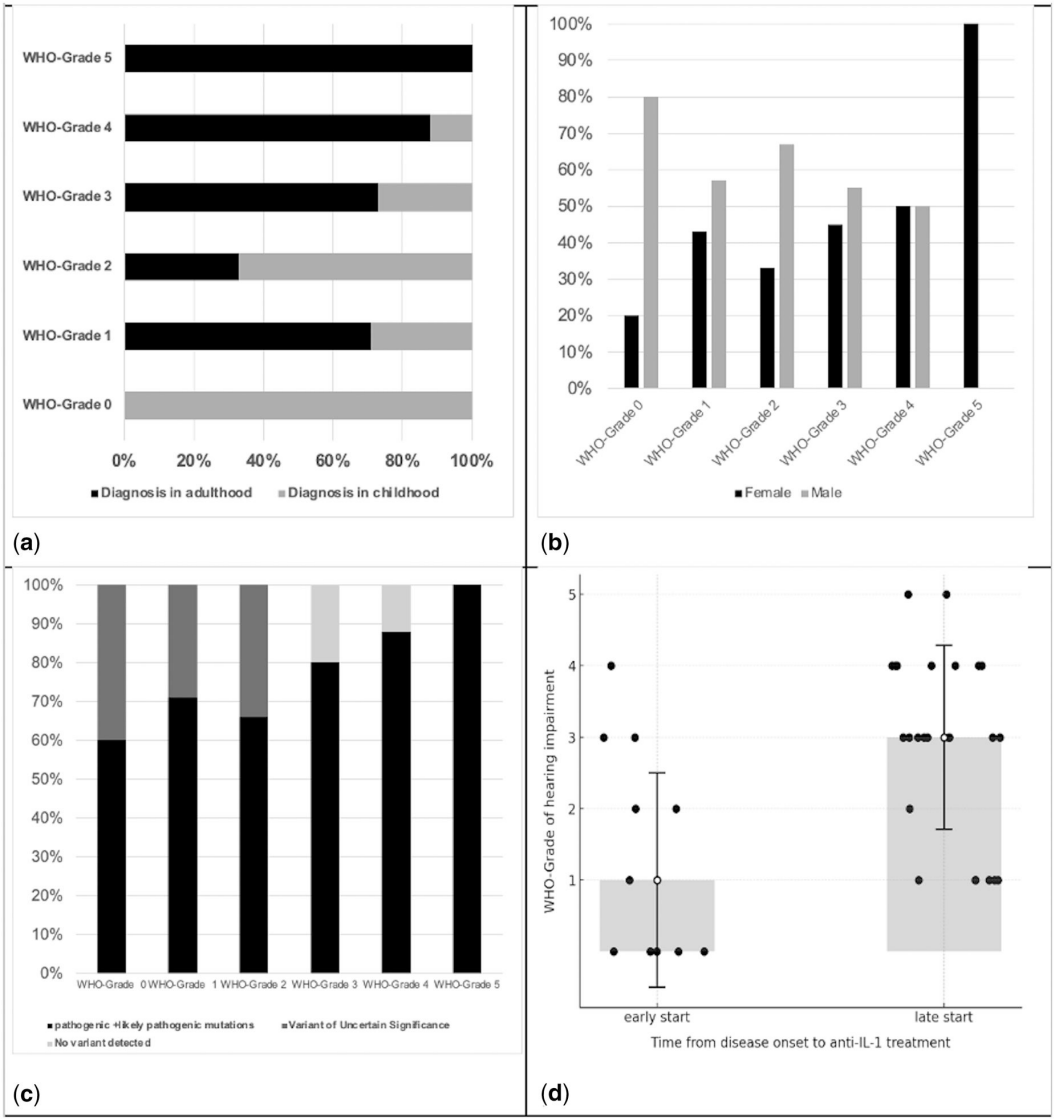
Association of patient- and treatment-related factors with WHO grade of hearing impairment in CAPS patients. (**a**) Hearing impairment in relation to age at diagnosis. At the last follow-up, the distribution of WHO Grades of hearing impairment differed significantly according to age at diagnosis (*P* = 0.04). All individuals with normal hearing (WHO Grade 0) had been diagnosed in childhood. In contrast, the majority of patients with moderate to profound hearing loss (WHO Grades 3–5) were diagnosed in adulthood. (**b**) Hearing impairment in relation to gender. At the last follow-up, normal hearing (WHO Grade 0) was observed in 20% of female patients and 80% of male patients. All patients with profound hearing loss (WHO Grade 5) were female. Although this sex-specific trend was notable, it did not reach statistical significance (*P* = 0.12). (**c**) Hearing impairment and NLRP3 variant pathogenicity. At last follow-up, patients with pathogenic or likely pathogenic mutations tended to have more severe WHO grade hearing impairment but the difference was not statistically significant (*P* = 0.072). (**d**) Hearing impairment and time to anti-IL1 treatment. At last follow-up, there was a trend towards higher-grade hearing impairment associated with late treatment start (*P* = 0.05)

At last follow-up, this association persisted (*χ*^2^, *P* = 0.04); all with normal hearing were childhood diagnoses, while most with moderate to profound loss were diagnosed in adulthood. Correlation remained significant (*r* = −0.45, 95% CI −0.67 to −0.17, *P* = 0.006) (Fig. 1a).

#### Gender

At baseline, a higher proportion of females had more severe hearing impairment compared with males. Among those with normal hearing, 40% were female and 60% male. The proportion of females with severe and profound hearing loss was 56% and 100%, respectively. However, this distribution was not statistically significant (*χ*^2^ test, *P* = 0.18; mean difference = 0.73, 95% CI −0.31 to 1.76, *P* = 0.163).

At last follow-up, 20% of female and 80% of male patients had normal hearing. The distribution of mild to severe hearing loss was similar between sexes, while all patients with profound hearing loss were female. Again, no significant difference was found (*χ*^2^ test, *P* = 0.12; mean difference = 0.78, 95% CI −0.24 to 1.79, *P* = 0.130) (Fig. 1b).

#### NLRP3 variant pathogenicity

At baseline, severe and profound hearing impairment was primarily associated with pathogenic or likely pathogenic variants (89% and 100%, respectively). A similar trend was observed at last follow-up, with 88% of those with severe hearing impairment and 100% of those with profound hearing impairment carrying pathogenic or likely pathogenic variants. In total, 21 patients with moderately severe, severe or profound hearing impairment (WHO grades 3, 4 and 5) were identified. Among these, 43% carried the likely pathogenic E311K variant, 24% the pathogenic T348M variant, 10% the pathogenic A439V variant and 4% the R262W variant. Nineteen percent had no detectable NLRP3 variant (Fig. 1c).

#### Time to treatment

At baseline, hearing impairment differed significantly based on the timing of anti-IL-1 treatment initiation (*χ*^2^ test, *P* = 0.013). In the early treatment group, 100% of patients had normal hearing, whereas 72% of those who started treatment late had moderately severe, severe or profound impairment. This was supported by a significant difference in hearing thresholds (mean difference = −1.47, 95% CI −2.49 to −0.44, *P* = 0.007).

At last follow-up, all patients in the early treatment group maintained normal hearing. In contrast, none in the late-start group had normal hearing, while 88% had severe and 100% had profound impairment (*χ*^2^ test, *P* = 0.05). The difference in hearing thresholds remained significant (mean difference = −1.56, 95% CI −2.55 to −0.56, *P* = 0.003) (Fig. 1d).

### Therapy escalation due to progressive hearing loss

A total of 10 out of 36 CAPS patients (28%)—including 5/6 with severe and 5/30 with moderate phenotype—required anti-IL-1 therapy escalation due to progression of sensorineural hearing loss. The group comprised seven females and three males. Eight patients carried pathogenic or likely pathogenic NLRP3 variants; two had a VUS. The mean time to progression was 5.4 years (range: 1–10.2).

At the time of progression, nine patients were receiving canakinumab and one was on anakinra. Three patients showed early progression with partially controlled disease activity, accompanied by increases in inflammatory markers and clinical symptoms (fatigue, rash, arthritis).

Among the seven with late progression, contributing factors included long-term treatment non-compliance (*n* = 4), puberty (*n* = 2) and pregnancy-associated non-compliance (*n* = 1). The pregnancy case also showed elevated inflammation and systemic symptoms, while the patient with progression during puberty exhibited CNS involvement and abnormal laboratory findings. Median non-compliance duration was four years (range: 2–7), except for the pregnancy case (9 months).

Following therapy escalation, six patients improved and four remained stable. Of the stable patients, three (75%) had a severe phenotype, all treated with dose escalation. Among those who improved, four (67%) had a moderate and two (33%) a severe phenotype; one responded to dose increase, and one required the addition of anakinra (2 mg/kg/day) to ongoing canakinumab (Supplementary Table S1, available at *Rheumatology* online).

## Discussion

### Overall findings

This study provides a comprehensive analysis of risk factors and real-life barriers influencing sensorineural hearing loss in the long-term treatment of patients with CAPS. The findings emphasize the critical importance of early diagnosis and timely intervention. The severity of hearing loss was found to be closely associated with specific risk factors. Anti-IL-1 therapies demonstrated significant efficacy in stabilizing hearing loss over a longer period and, in some patients, improving it. However, in some patients, auditory deterioration occurred despite anti-IL-1 treatment. In the early stages, progression was associated with partially controlled disease activity, whereas late-stage progression was linked to treatment non-compliance and factors such as pregnancy and puberty. These represent real-life barriers to optimal management and underscore the importance of individualized treatment adjustments and long-term monitoring including therapy adherence.

The severity of hearing loss was found to be closely associated with specific risk factors. Patients diagnosed in adulthood, those starting treatment late and individuals carrying pathogenic or likely pathogenic mutations exhibited higher WHO grades of hearing impairment at baseline and last follow-up. We previously reported that the severity of hearing loss in CAPS progressively increases over time [12]. Additionally, the extent of hearing loss deteriorates with advancing age [4]. Attention to the severity of the CAPS phenotype remains critical, as more intensive treatment is often required to achieve remission and/or prevent progression of hearing loss in those with severe CAPS [16, 17]. Furthermore, our study emphasized the need to test high-frequency (6–8 kHz) thresholds in routine CAPS assessments for early detection of hearing impairment [4], since this could be a predictor for further progression personalized monitoring, early intervention and targeting disease-related and modifiable risk factors, may help improve long-term outcomes.

### Implications for research

Progressive sensorineural hearing loss in CAPS was detected both in the early and late stages, even after years of anti-IL-1 treatment. Early progression was associated with evidence of only partially controlled disease activity. In contrast, late progression was associated with modifiable factors, such as non-compliance, pregnancy-associated non-compliance, infections and puberty resulting in increased disease activity. These data highlight the challenges of implementing T2T strategies for a lifelong illness in routine clinical practice. Guidance on optimal monitoring in lifelong CAPS are limited. Romano *et al.* emphasized the importance of standardized monitoring, particularly the necessity of regular audiological assessments [6].

### Implications for practice

Recommendations on optimal CAPS monitoring during pregnancy and puberty remain limited. A recent review of Davidson *et al.* provided insights from pregnancies in women with Familial Mediterranean Fever. Some reports suggested reduced disease activity during pregnancy, while others indicated comparable disease severity and episode frequency to non-pregnant periods [15]. Long-term management strategies should be refined by incorporating modified risk profiles such as puberty, pregnancy and breastfeeding.

### Strengths and limitations

This study has several limitations. It is a single-centre observational study with a limited sample size and therefore missing data. However, institutional protocols together with the electronic patient record system ensures data consistency and maintains quality. Despite the exceptionally rare occurrence of CAPS, the study provides highly relevant information regarding the clinical management of this orphan disease. Another important limitation is that these findings may not be generalizable to other similar populations, given the specific characteristics of the study cohort and setting.

## Conclusion

In conclusion, early diagnosis, timely intervention and a refined T2Tapproach are essential for the lifelong management of CAPS and its complications including sensorineural hearing loss. Individualized care addressing the identified risk factors and continuous monitoring are crucial to improving long-term outcomes.

## Supplementary Material

keaf355_Supplementary_Data

## Data Availability

Data are available from the corresponding author upon reasonable request.
