# Supplementary material for: Sensorineural hearing loss in anti-interleukin-1 treated CAPS patients: risk factors and real-life barriers—an observational study
Source: Rheumatology (Oxford). 2025 Aug 1;64(12):6359–65. doi: 10.1093/rheumatology/keaf355 (PMC12671876; doi:10.1093/rheumatology/keaf355)
Supplement: keaf355_Supplementary_Data [file keaf355_supplementary_data.docx]

**Supplementary Table S1: CAPS (Cryopyrin-Associated Periodic Syndromes) patients requiring therapy escalation due to progressive sensorineural hearing loss.**

| Gender and age at diagnosis  in years | CAPS  phenotype and  *NLRP3*  variant | WHO grade of hearing impairment at baseline | Anti-IL1 treatment type and duration at progression | WHO grade of hearing impairment at escalation | Type of treatment escalation | WHO grade of hearing impairment at last follow-up | Indication for treatment escalation |
| --- | --- | --- | --- | --- | --- | --- | --- |
| **Early progression (≤ 3 years of treatment)** | | | | | | | |
| M, 11 | severe CAPS,  H358R, likely pathogenic | 0 | canakinumab  **1 year** | 2 | dose increase (canakinumab) | 0 | disease activity |
| M, 60 | severe CAPS,  T348M, pathogenic | 3 | anakinra  **1 year** | 4 | dose increase  (anakinra) | 4 | disease activity |
| F, 4 | moderate CAPS  E311K, likely pathogenic | 1 | canakinumab  **3 years** | 2 | dose increase (canakinumab) | 1 | disease activity |
| **Late progression (>3 years of treatment)** | | | | | | | |
| F, 19 | moderate CAPS,  E311K, likely pathogenic | 3 | canakinumab  **5 years** | 4 | dose increase  (canakinumab) | 3 | noncompliance pregnancy |
| F, 5 | severe CAPS,  N654S, VUS | 0 | canakinumab  **5 years** | 1 | dose increase (canakinumab) | 1 | noncompliance |
| F, 5 | moderate CAPS  Y859S, likely pathogenic | 0 | canakinumab  **7 years** | 1 | dose increase (canakinumab) | 0 | noncompliance |
| F, 5 | moderate CAPS  Y859S, likely pathogenic | 0 | canakinumab  **8 years** | 1 | dose increase (canakinumab) | 0 | noncompliance |
| M, 6 | severe CAPS  M664T, pathogenic | 0 | canakinumab  **9 years** | 1 | combination (canakinumab + anakinra) | 0 | puberty |
| F, 12 | moderate CAPS  T348M, pathogenic | 1 | canakinumab  **9 years** | 2 | dose increase (canakinumab) | 2 | noncompliance |
| F, 3 | severe CAPS  V198M, VUS | 0 | canakinumab  **10 years** | 1 | dose increase (canakinumab) | 1 | puberty  infections |

Legend: WHO Grade 0 (≤ 25 dB, no hearing impairment), Grade 1 (26–40 dB, mild hearing impairment), Grade 2 (31–60 dB for children, 41–60 dB for adults, moderate hearing impairment), Grade 3 (61–80 dB, severe hearing impairment), and Grade 4 (≥ 81 dB, profound hearing impairment). VUS: Variant of Uncertain Significance, WHO: World Health Organisation. Bold text is used to emphasize findings considered particularly relevant or important for clinical interpretation. These highlighted results are intended to draw attention to the most meaningful aspects of the data.
